# Isoflurane Attenuates Cerebral Ischaemia–Reperfusion Injury via the TLR4-NLRP3 Signalling Pathway in Diabetic Mice

**DOI:** 10.1155/2022/2650693

**Published:** 2022-04-04

**Authors:** Ya-Jun Zhang, Wen-Jing Guo, Zi-Yuan Tang, Hong-Bin Lin, Pu Hong, Jing-Wei Wang, Xuan-Xuan Huang, Feng-Xian Li, Shi-Yuan Xu, Hong-Fei Zhang

**Affiliations:** ^1^Department of Anesthesiology, Zhujiang Hospital of Southern Medical University, Guangzhou, Guangdong, China; ^2^Department of Anesthesiology, Dalian Municipal Maternal and Child Health Care Hospital, Dalian, Liaoning, China; ^3^Department of Anesthesiology, The First Affiliated Hospital of Shantou University Medical College, Shantou, Guangdong, China

## Abstract

Ischaemic stroke is a severe disease worldwide. Restoration of blood flow after ischaemic stroke leads to cerebral ischaemia–reperfusion injury (CIRI). Various operations, such as cardiac surgery with deep hypothermic circulatory arrest, predictably cause cerebral ischaemia. Diabetes is related to the occurrence of perioperative stroke and exacerbates neurological impairment after stroke. Therefore, the choice of anaesthetic drugs has certain clinical significance for patients with diabetes. Isoflurane (ISO) exerts neuroprotective and anti-neuroinflammatory effects in patients without diabetes. However, the role of ISO in cerebral ischaemia in the context of diabetes is still unknown. Toll-like receptor 4 (TLR4) and NOD-like receptor pyrin domain-containing protein 3 (NLRP3) inflammasome activation play important roles in microglia-mediated neuroinflammatory injury. In this study, we treated a diabetic middle cerebral artery occlusion mouse model with ISO. We found that diabetes exacerbated cerebral ischaemia damage and that ISO exerted neuroprotective effects in diabetic mice. Then, we found that ISO decreased TLR4-NLRP3 inflammasome activation in microglia and the excessive autophagy induced by CIRI in diabetic mice. The TLR4-specific agonist CRX-527 reversed the neuroprotective effects of ISO. In summary, our study indicated that ISO exerts neuroprotective effects against the neuroinflammation and autophagy observed during diabetic stroke via the TLR4-NLRP3 signalling pathway.

## 1. Introduction

Stroke is a severe disease with a poor prognosis, and it places a heavy burden on society and the economy [1]. Approximately, 30 million people suffer from stroke every year. Among patients with stroke, the proportion of patients with ischaemic stroke is as high as 80% [2], and the incidence of perioperative stroke reaches 7% [3]. Notably, perioperative stroke occurs during various neurosurgical operations and cardiac surgeries, such as deep hypothermic circulatory arrest and carotid endarterectomy.

Diabetes increases the risk of perioperative ischaemic stroke [4]. In addition, diabetes exacerbates vascular injury and inflammation, which worsen stroke outcomes [5]. Consequently, it is particularly important to determine the molecular mechanism underlying ischaemic stroke in the context of diabetes and to seek effective treatment strategies for these patients during surgery. A clinical study showed that inhaled anaesthetics reduce the incidence of perioperative ischaemic stroke [6]. Isoflurane (ISO) is a common inhaled anaesthetic that is used in the clinic. Notably, ISO exerts anti-inflammatory and neuroprotective effects on ischaemic stroke in patients without diabetes [7–9], but whether ISO exerts neuroprotective effects against ischaemic stroke in the context of diabetes remains unknown.

Effectively protecting dying neurons against cerebral ischaemia–reperfusion injury (CIRI) is challenging. Among the potential pathological mechanisms underlying CIRI, neuroinflammation plays a prominent role during the injury process [10]. Toll-like receptor 4 (TLR4) is involved in the inflammatory response [11]. Notably, previous studies demonstrated that ISO inhibits TLR4 activation during ischaemic stroke in patients without diabetes [12–14]. Additionally, TLR4 inhibition improves neurological function after stroke in diabetic rats [15]. Therefore, we hypothesized that TLR4 may be a target through which ISO exerts its neuroprotective effect in patients with diabetic stroke. The NOD-like receptor pyrin domain-containing protein 3 (NLRP3) inflammasome is an inflammatory activator that is closely related to TLR4 [16, 17]. Our previous study confirmed that the activated NLRP3 inflammasome participates in the mechanism underlying CIRI in diabetic mice [18]. However, it is still rather unclear whether NLRP3 inflammasome activation is regulated by TLR4 during CIRI in the context of diabetes. Moreover, autophagy also participates in the process of stroke. CIRI induces excessive autophagy, which exacerbates brain injury. Inhibition of excessive autophagy exerts neuroprotective effects [19]. TLR4 activation regulates autophagy and affects neuronal function [20]. However, few studies have described the mechanism underlying the occurrence autophagy after stroke in the context of diabetes.

Overall, we hypothesized that inhibition of TLR4-NLRP3 inflammatory signalling is closely associated with the neuroprotective effects of ISO in diabetic CIRI. We used C57BL/6J mice to establish a diabetic ischaemic stroke model. Then, we treated these mice with ISO to determine whether ISO has neuroprotective properties and explore the possible underlying mechanism in diabetic mice with CIRI. Our results indicated that ISO attenuated CIRI in diabetic mice by inhibiting TLR4-NLRP3 inflammasome activation and autophagy. Additionally, our work identified the pathway by which brain damage occurs after diabetic ischaemic stroke and provided potential therapeutic targets for these patients.

## 2. Materials and Methods

### 2.1. Animals

The animal experiments were approved by the Animal Experiment Ethics Committee of Zhujiang Hospital of Southern Medical University. C57BL/6J mice (4 weeks of age, weighing 15-19 g, male) were purchased from Guangdong Medical Animal Experiment Center (Guangzhou, China).

### 2.2. Type 2 Diabetes Mellitus (T2DM) Mouse Model

Mice were fed a high-fat diet (4 weeks-12 weeks). The body weights and blood glucose levels were monitored. The mice were intraperitoneally injected with 100 mg/kg streptozotocin (STZ, Sigma, St. Louis, MO, USA) at 8 weeks of age. The fasting plasma glucose (FPG) levels were monitored. The T2DM model was considered to have been successfully established when the FPG level ≥ 10.0 mmol/L at 12 weeks of age, and the mice that met this criterion were used for follow-up experiments. Nondiabetic mice (non-DM) were fed a normal diet and were not injected with STZ [21].

### 2.3. Focal Cerebral Ischaemia Model

Transient middle cerebral artery occlusion (MCAO) was performed to simulate stroke according to the protocol described in our previous study [18]. In short, the mice were anaesthetized with ISO (RWD Life Sciences Co., Ltd., Shenzhen, China). A nylon monofilament with a blunt head (#0625-0627 depending on the mouse body weight, Yushun Biotechnology Co., Pingdingshan, China) was inserted to interrupt the blood supply in the right middle cerebral artery (MCA), and the nylon monofilament was maintained in this position for 60 min. Then, the monofilament was removed to induce reperfusion. The mice were sacrificed after 24 h. The mice in the sham group underwent a similar surgery except that a monofilament was not inserted. The mice were kept warm during and after the operation, and the rectal temperature was monitored and maintained between 36.5 and 37.5°C.

### 2.4. Laser Speckle Contrast Imaging (LSCI)

The mice were anaesthetized with ISO, the skin on top of the skull was disinfected, and a sagittal incision was made along the midline. A laser speckle probe (SIM BFI-HR Pro, Xunwei Technology Co., Wuhan, China) was placed at the center of the sagittal incision. CBF was measured before middle cerebral artery occlusion (MCAO) (baseline), immediately after MCA blocking, and at the beginning of reperfusion. The successful establishment of MCAO was confirmed using criteria described in previous studies [22], which included that the area of MCA CBF decreased by more than 50% after occlusion.

### 2.5. Drug Administration and Experimental Groups

The mice in the ISO [ISO(+)] group were administered 1.5% ISO in oxygen (O_2_) by inhalation for 60 min. The control [ISO(−)] group inhaled O_2_. The mice in both groups were then randomly subjected to MCAO or sham operation. Intraperitoneal injection of 3 mg/kg TAK-242 (HY-11109, MCE, USA) or 0.25 mg/kg CRX-527 (tlrl-crx527, InvivoGen, Hong Kong) was performed before reperfusion according to previous studies and the manufacturer's instructions [23, 24]. In short, the mice were divided into eleven groups: (1) non-DM sham group, (2) non-DM MCAO group, (3) DM sham group, (4) DM MCAO group, (5) DM MCAO with ISO [DM MCAO ISO(+)] group, (6) DM MCAO without ISO [DM MCAO ISO(−)] group, (7) DM MCAO with TAK-242 treatment [DM MCAO TAK-242] group, (8) DM sham with ISO and vehicle treatment [DM ISO(+) sham vehicle] group, (9) DM sham with ISO and CRX-527 treatment [DM ISO(+) sham CRX-527] group, (10) DM MCAO with ISO and vehicle treatment [DM ISO(+) MCAO vehicle] group, and (11) DM MCAO with ISO and CRX-527 treatment [DM ISO(+) MCAO CRX-527] group.

### 2.6. Neurobehavioural Assessment

Behavioural impairment was scored according to classical scoring criteria (Table 1) [25].

Mice with a neurobehaviour score of 0 (except those in the sham group) or 4 and those that died within 24 h after the operation were excluded.

### 2.7. 2,3,5-Triphenyltetrazolium Chloride (TTC) Staining

The brains were sliced and immersed in 2% TTC (bccc4696, Sigma, USA). The infarction size was calculated in accordance with the following formula: cerebral infarction volume percentage = (contralateral brain volume − ipsilateral normal brain volume)/contralateral brain volume × 100%.

### 2.8. Western Blotting

Right hemisphere tissues were collected and prepared. The antibody information is listed in Supplementary Table S1. The bands were developed by a chemiluminescent reagent (Millipore, WBKLS0500, MA, United States). Relative protein expression was determined by densitometric analysis with ImageJ software (1.8.0, NIH, USA).

### 2.9. Immunohistochemistry and Immunofluorescence

Brain sections (4 *μ*m) were prepared, incubated with primary antibodies (Supplementary Table S1), and then incubated with biotin-labelled goat anti-mouse/rabbit IgG (sp-9000-6 mL, ZSGB, China) or immunofluorescence secondary antibodies (Supplementary Table S1). Images were captured by using an orthogonal microscope (dm2500, Leica, Germany) or fluorescence microscope (TS100, Nikon, Japan).

### 2.10. Statistical Analysis

The data are shown as the means ± SDs and were analysed using GraphPad Prism 7 (San Diego, CA, USA). Normality assessment was performed with the Kolmogorov–Smirnov test (Dallal-Wilkinson-Lillie correction). Student's *t*-test was used to compare data between two groups. Data from four groups were analysed using one-way or two-way ANOVA followed by the Tukey–Kramer post hoc test. Differences were considered statistically significant if *P* < 0.05.

## 3. Results

### 3.1. Diabetes Exacerbated Cerebral Ischaemia-Reperfusion Injury

**Figure 1:**
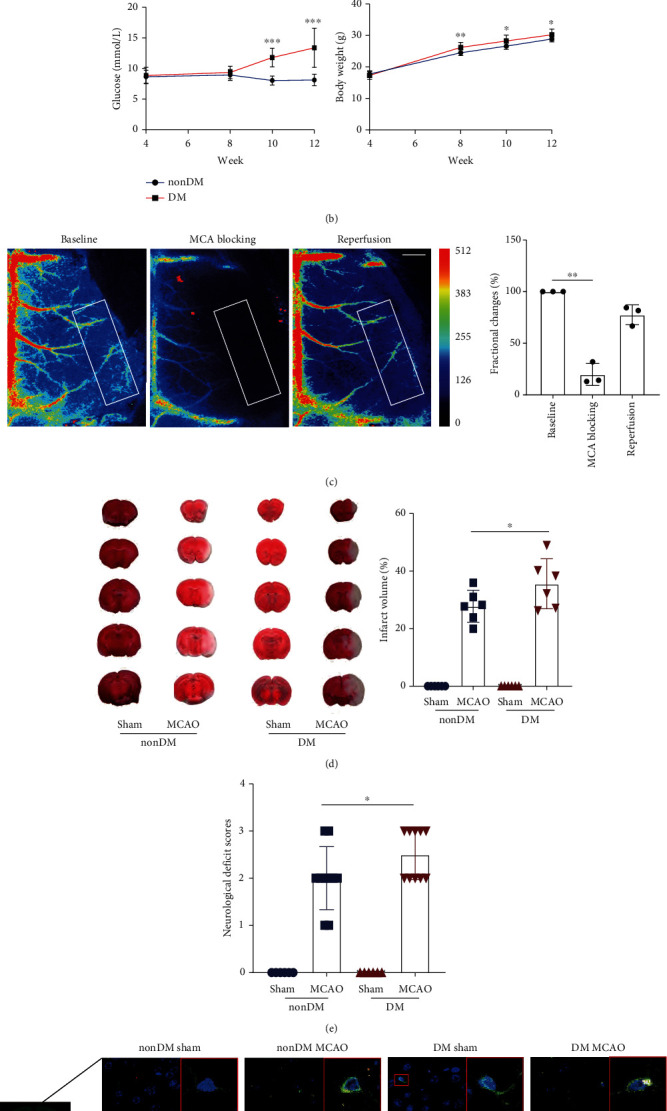
Cerebral ischaemia-reperfusion induced more severe damage under diabetes. (a) Diagram of the T2DM mouse model establishment. (b) Continuous measurement of FPG levels and weight in diabetic mice and nondiabetic mice starting at high-fat diet feeding (4 weeks of age). (c) Left, representative images of LSCI measurement showing CBF changes during MCAO model construction (white frames refer to the MCA supply area, scale bar: 1 mm). Right, the CBF fractional changes at the timepoint of baseline, MCA blocking and reperfusion (*n* = 3). (d) TTC-stained slices from non-DM mice and DM mice in the sham and MCAO groups, and the bar graph shows ipsilateral infarct size of each group (*n* = 6 in each group). (e) The neurological deficit scores of the non-DM mice and DM mice in the sham and MCAO groups (*n* = 6 in the non-DM and DM sham groups, *n* = 10 in the non-DM and DM MCAO groups). (f) Representative immunofluorescence images that microglia (IBA-1, green) expressed TLR4 (red) and NLRP3 (white) colocalization (scale bar: 9.94 *μ*m, magnification: 640x). ^∗^*P* < 0.05; ^∗∗^*P* < 0.01; ^∗∗∗^*P* < 0.001.

To assess the differences in neuroinflammatory injury after CIRI between non-DM mice and DM mice, we established a T2DM mouse model (Figure 1(a)). The FPG levels and body weights were significantly increased in the diabetic mice after 8 weeks of age (Figure 1(b)).

The cerebral blood flow (CBF) on the ipsilateral side decreased by more than 70% relative to baseline in the MCAO group after monofilament insertion (Figure 1(c)), which was consistent with our previous results of monitoring CBF by laser Doppler [18]. TTC staining revealed significant ischaemic brain damage in both the non-DM and DM MCAO groups (Figure 1(d)). Compared with the non-DM MCAO group, the mice in the DM MCAO group had larger cerebral infarction and worse neurobehavioural scores (Figures 1(d) and 1(e)). Additionally, the DM MCAO group had more TLR4-NLRP3-IBA-1 colocalization fluorescence signal than the non-DM MCAO group (Figure 1(f)). These findings indicated that CIRI caused more severe brain damage and worse outcomes in mice with diabetes. This effect is likely related to TLR4-NLRP3 activation.

### 3.2. Cerebral Ischaemia-Reperfusion Activated the TLR4-NLRP3 Inflammasome and Autophagy Pathways in Diabetic Mice

**Figure 2:**
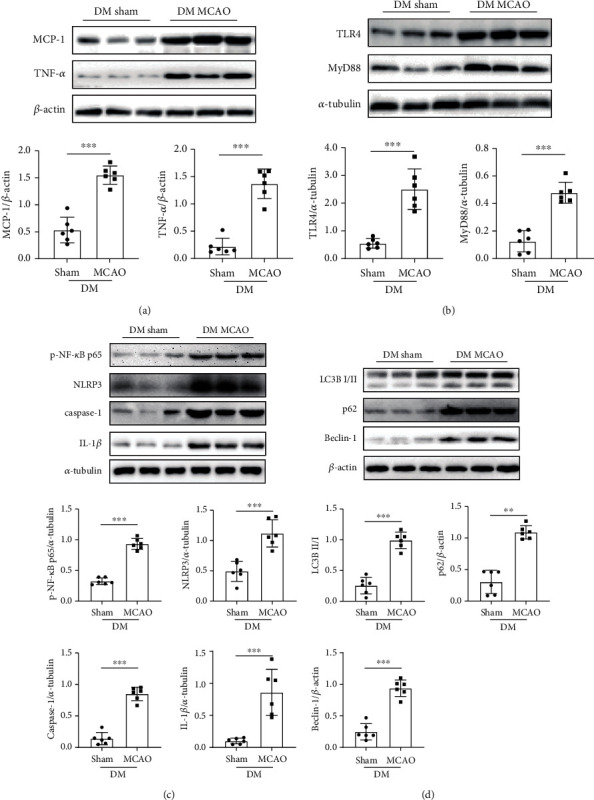
Cerebral ischaemia-reperfusion increased inflammatory factors and activated the TLR4, NLRP3 inflammasome, and autophagy pathways in diabetic mice. (a) Top, western blot images of MCP-1 and TNF-*α* in DM mice in the sham and MCAO groups (*n* = 3 in each group). Bottom, the expression of MCP-1 (left) and TNF-*α* (right, *n* = 6 in each group). (b) Top, western blot images of TLR4 and MyD88 (*n* = 3 in each group). Bottom, the expression of TLR4 (left) and MyD88 (right, *n* = 6 in each group). (c) Top, western blot images of p-NF-*κ*B p65 and NLRP3 inflammasome-related proteins (*n* = 3 in each group). Middle, the expression of p-NF-*κ*B p65 (left) and NLRP3 (right). Bottom, the expression of caspase-1 (left) and IL-1*β* (right, *n* = 6 in each group). (d) Top, western blot images of autophagy-related proteins (*n* = 3 in each group). Middle, the expression of LC3B (left) and p62 (right). Bottom, the expression of Beclin-1 (*n* = 6 in each group). ^∗∗^*P* < 0.01; ^∗∗∗^*P* < 0.001.

Compared with the non-DM MCAO group, the DM MCAO group exhibited increased TLR4 activation, nuclear factor-*κ*B (NF-*κ*B) phosphorylation, and NLRP3 inflammasome-related protein expression (Supplementary Figure S1). Subsequently, we chose diabetic mice to explore the potential molecular mechanism underlying CIRI, which has a worse prognosis. We found that the expression of the proinflammatory factors monocyte chemotactic protein-1 (MCP-1) and tumour necrosis factor-*α* (TNF-*α*) was significantly increased in the DM MCAO group compared with the DM sham group (Figure 2(a)). This finding indicated neuroinflammatory activation after CIRI. The DM MCAO group exhibited greater TLR4 activation after CIRI compared to the DM sham group (Figure 2(b)). Similarly, NF-*κ*B phosphorylation and NLRP3 inflammasome-related protein expression were significantly increased in the DM MCAO group (Figure 2(c)). Additionally, TLR4 is a sensor of autophagy [26]. Autophagy was significantly activated in the DM MCAO group (Figure 2(d)). These results suggested that CIRI activated the TLR4-NLRP3 inflammasome and autophagy pathways in mice with diabetes.

### 3.3. Cerebral Ischaemia–Reperfusion Activated TLR4-Positive Microglia in Diabetic Mice

**Figure 3:**
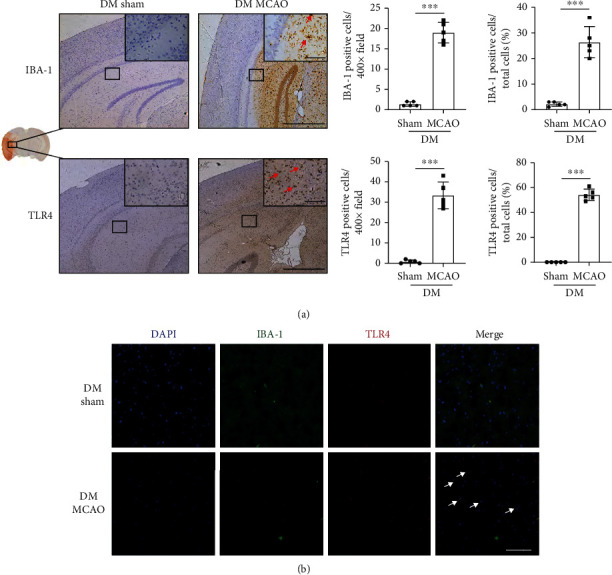
Cerebral ischaemia-reperfusion enhanced TLR4-positive microglial recruitment under diabetes. (a) Top, representative immunohistochemical images of microglia in the ischaemic penumbra (left, large picture scale bar: 800 *μ*m, magnification: 50x; small picture scale bar: 100 *μ*m, magnification: 400x). Arrows, microglia labelled with IBA-1. Bar graphs showing the total number and the ratio of IBA-1-positive cells in diabetic sham and MCAO groups (*n* = 5 in each group). Bottom, representative immunohistochemical images of TLR4 staining in the ischaemic penumbra (left, large picture scale bar: 800 *μ*m, magnification: 50x; small picture scale bar: 100 *μ*m, magnification: 400x). Arrows, TLR4 positive cells. Bar graphs (right) showing the total number and the ratio of TLR4-positive cells (*n* = 5 in each group). (b) Representative immunofluorescence images that microglia (IBA-1) expressed TLR4 (scale bar: 100 *μ*m, magnification: 200x). Arrows, IBA-1 and TLR4 copositive cells. ^∗∗∗^*P* < 0.001.

Microglia are indispensable for the inflammatory response after stroke, and NLRP3 is mainly expressed in microglia [27]. Therefore, we assessed the microglia in the DM mice after stroke. IBA-1 is a marker of microglia. We found that microglia were recruited to the ischaemic penumbra in the DM MCAO group (Figure 3(a)). Additionally, the DM MCAO group had more TLR4-positive cells (Figure 3(a)). Furthermore, these cells were mostly IBA-1-positive microglia (Figure 3(b)). These findings indicated that CIRI increased TLR4-positive microglial recruitment to the ischaemic penumbra.

### 3.4. ISO Alleviated CIRI and Inhibited TLR4-NLRP3 Inflammasome Pathway Activation in Diabetic Mice

**Figure 4:**
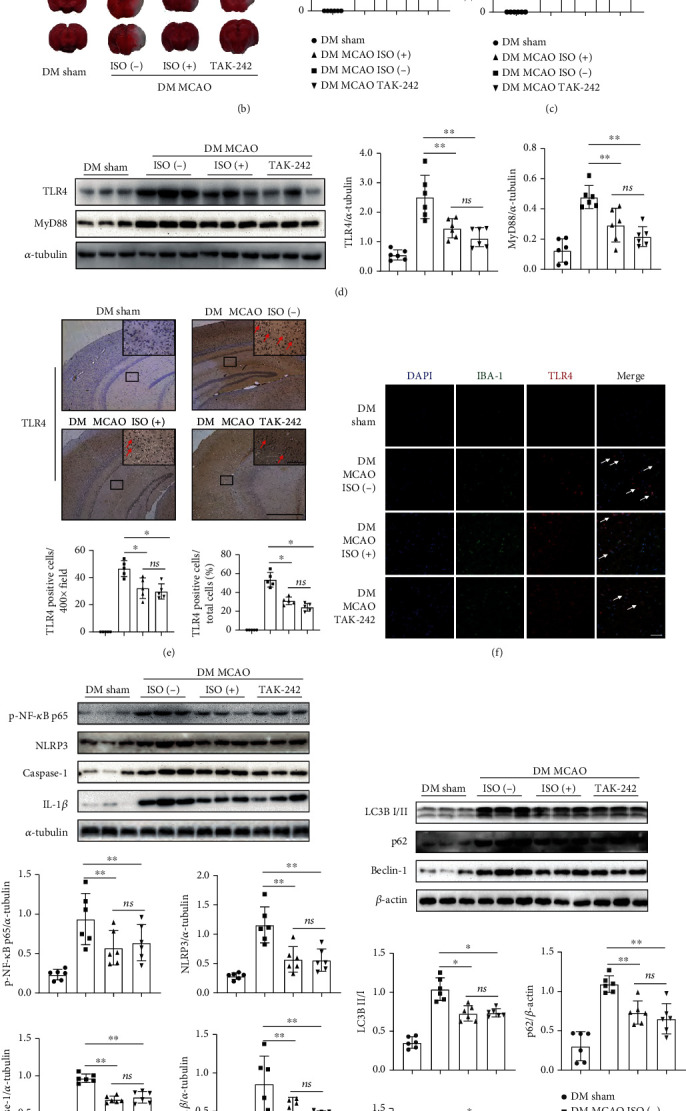
ISO suppressed the TLR4-NLRP3 inflammasome pathway in diabetic CIRI. (a) Diagram of the protocol of the ISO. (b) Left, TTC-stained slices from the sham, MCAO ISO(−), MCAO ISO(+), and MCAO TAK-242 groups in DM mice. Right, the ipsilateral infarct volume of each group (*n* = 6). (c) The bar graph shows the neurological scores (*n* = 6 − 10). (d) Left, western blot images of TLR4 and MyD88 (*n* = 3 in each group). Bar graphs showing TLR4 (middle) and MyD88 (right) expression (*n* = 6). (e) Top, representative immunohistochemical images of TLR4 staining in the ischaemic penumbra (large picture scale bar: 800 *μ*m, magnification: 50x, small picture scale bar: 100 *μ*m, magnification: 400x). Arrows, TLR4-positive cells. Bottom, bar graphs showing the total number (left) and the ratio of TLR4-positive cells (right, *n* = 5). (f) Representative immunofluorescence images that microglia expressed TLR4 (scale bar: 100 *μ*m, magnification: 200x). Arrows, IBA-1 and TLR4 copositive cells. (g) Top, western blot image of p-NF-*κ*B p65, NLRP3 inflammasome-related proteins (*n* = 3). Middle, the expression of p-NF-*κ*B p65 (left) and NLRP3 (right) expression. Bottom, bar graphs showing caspase-1 (left) and IL-1*β* (right) expression (*n* = 6). (h) Top, western blot image of autophagy-related proteins (*n* = 3). Middle, the expression of LC3B (left) and p62 (right). Bottom, the expression of Beclin-1 (*n* = 6). ^∗^*P* < 0.05; ^∗∗^*P* < 0.01.

To investigate whether ISO exerts a neuroprotective effect against CIRI in diabetic mice, we pretreated diabetic mice with 1.5% ISO for 1 h before inducing MCAO (Figure 4(a)). The TLR4-specific inhibitor TAK-242 was used as a control. We found that the infarct volume and neurobehavioural scores were both decreased after ISO or TAK-242 treatment (Figures 4(b) and 4(c)). TLR4 and MyD88 protein expression was also significantly decreased in these groups (Figure 4(d)). Consistent with the protein results, the numbers of TLR4-positive cells in the penumbra were also significantly decreased, as were the numbers of TLR4-positive microglia (Figures 4(e) and 4(f)). These data show that ISO protected against CIRI in diabetic mice.

In further experiments, we focused on the effects of ISO on inflammatory factors and NLRP3 inflammasome-related proteins. We found that NF-*κ*B phosphorylation and NLRP3 inflammasome-related protein expression were decreased in the DM MCAO ISO(+) group and DM MCAO TAK-242 group (Figure 4(g)). This result indicated that ISO exerts its neuroprotective effect on diabetic stroke by inhibiting the TLR4-NLRP3 signalling pathway. Moreover, we found that autophagy-related protein expression was also significantly decreased in these groups (Figure 4(h)). These results demonstrated that ISO protects diabetic mice from stroke by inhibiting the TLR4-NLRP3 inflammasome-related inflammatory response and autophagy.

### 3.5. TLR4-NLRP3 Inflammasome Pathway Inhibition Was Indispensable for ISO Neuroprotection

**Figure 5:**
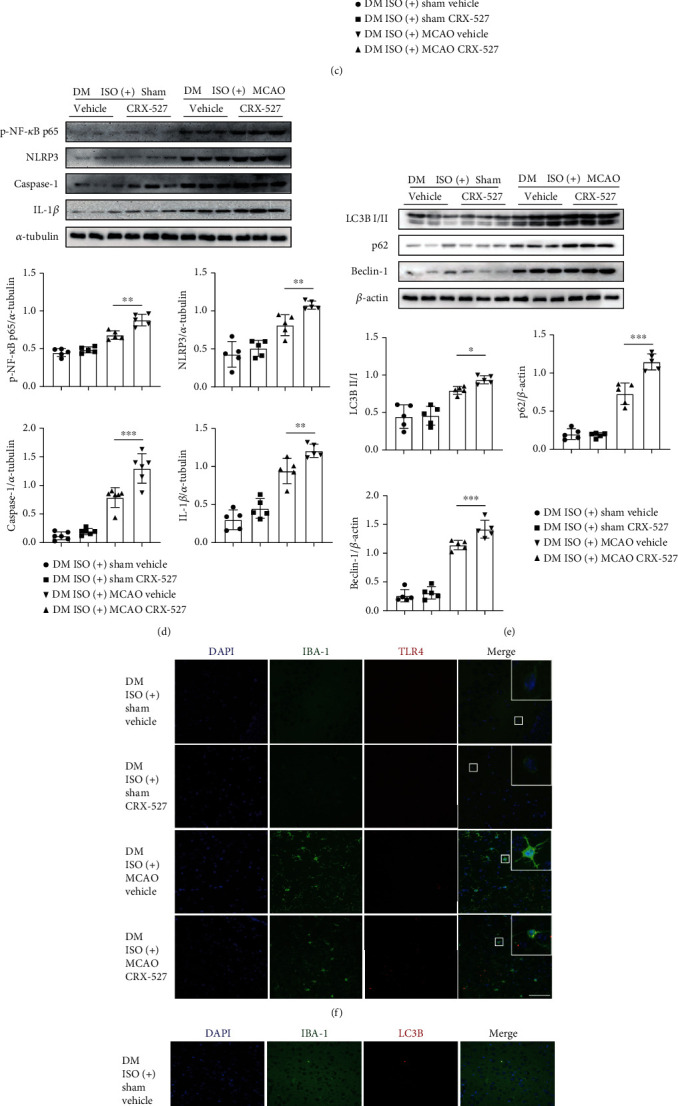
CRX-527 treatment reversed ISO neuroprotection in diabetic CIRI. (a) Left, TTC-stained slices from the ISO(+) sham vehicle, ISO(+) sham CRX-527, ISO(+) MCAO vehicle, and ISO(+) MCAO CRX-527 groups in DM mice. Right, the bar graph shows the ipsilateral infarct size of each group (*n* = 3 − 6 in each group). (b) The bar graph shows the neurological scores (*n* = 3 − 10 in each group). (c) Left, western blot image of TLR4 and MyD88 (*n* = 3 in each group). Middle and right, bar graphs showing TLR4 (middle) and MyD88 (right) expression (*n* = 5 in each group). (d) Top, western blot image of p-NF-*κ*B p65, NLRP3 inflammasome-related proteins (*n* = 3 in each group). Middle, bar graphs showing p-NF-*κ*B p65 (left) and NLRP3 (right) expression (*n* = 5 in each group). Bottom, bar graphs showing caspase-1 (left, *n* = 6 in each group) and IL-1*β* (right, *n* = 5 in each group) expression. (e) Top, western blot image of autophagy-related proteins (*n* = 3 in each group). Middle, the expression of LC3B (left) and p62 (right). Bottom, the expression of Beclin-1 (*n* = 5 in each group). (f) Representative immunofluorescence images that microglia expressed TLR4 (scale bar: 100 *μ*m, magnification: 400x). (g) Representative immunofluorescence images that microglia expressed LC3B (scale bar: 100 *μ*m, magnification: 400x). ^∗^*P* < 0.05; ^∗∗^*P* < 0.01; ^∗∗∗^*P* < 0.001.

To further elucidate whether ISO neuroprotection is mediated by TLR4 inhibition, mice were administered a TLR4-specific agonist, namely, CRX-527. Compared with the group that received ISO alone, the DM ISO(+) MCAO CRX-527 group exhibited increased infarct volumes (Figure 5(a)) and neurobehavioural scores (Figure 5(b)), indicating that CRX-527 reversed the effect of ISO. Inevitably, TLR4, MyD88, p-NF-*κ*B p65, and NLRP3 inflammasome-related protein expression was significantly enhanced after CRX-527 administration (Figures 5(c) and 5(d)), suggesting that CRX-527 reactivated the TLR4-NLRP3 pathway. Additionally, autophagy-related protein expression was also elevated (Figure 5(e)). More IBA-1 and TLR4 double positive cells were observed in the DM ISO(+) MCAO CRX-527 group than in the control group (Figure 5(f)). Additionally, more IBA-1 and LC3B double positive cells were observed in the DM ISO(+) MCAO CRX-527 group than in the control group (Figure 5(g)). Our data thus indicated that CRX-527 activates the TLR4-NLRP3 inflammasome pathway and autophagy in microglia and reverses the neuroprotective effect of ISO in diabetes.

## 4. Discussion

**Figure 6:**
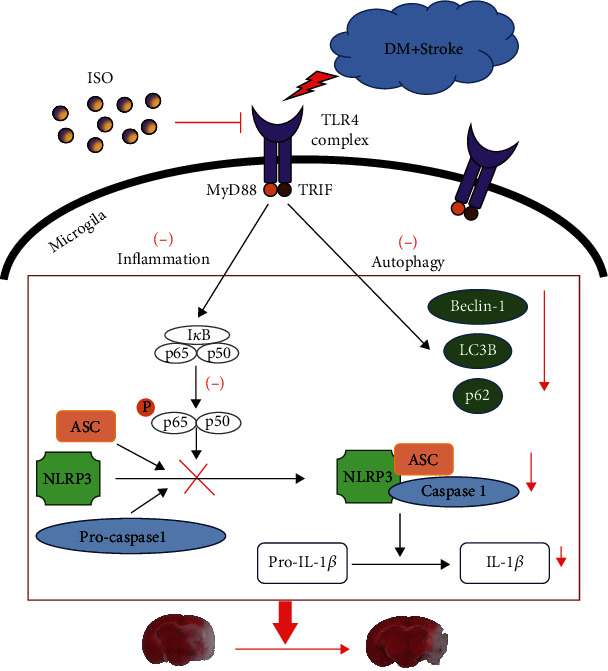
Scheme of the ISO neuroprotective mechanism in diabetic CIRI.Ischaemic stroke induced an inflammatory response and autophagy activation in diabetic mice. ISO inhibited TLR4 activation and downstream NF-*κ*B p65 phosphorylation, resulting in suppressing NLRP3 inflammasome formation. Finally, the proinflammatory factor IL-1*β* accumulation was descended. In addition, ISO alleviated the overactivation of autophagy. In summary, ISO attenuated CIRI and provided a neuroprotective effect.

In this work, ISO was found to exert a neuroprotective effect against CIRI in diabetic mice by inhibiting TLR4-NLRP3 inflammasome activation. First, we demonstrated that diabetes exacerbated CIRI and activated the inflammatory response and autophagy in diabetic mice with ischaemic stroke. Then, we confirmed that ISO reduced CIRI by decreasing the infarct volume and ameliorating neurological prognosis. Subsequently, we indicated that ISO inhibited TLR4-NLRP3 inflammasome activation and autophagy in microglia (Figure 6). In addition, we found that ISO-mediated neuroprotection could be reversed by a TLR4 agonist.

The transient middle cerebral artery occlusion (tMCAO) model is one of the models that mostly closely simulates human cerebral ischaemia-reperfusion injury, and it is used in most studies involving pathophysiological processes or neuroprotective agents [28]. To establish this model, we used a thread plug to block the middle cerebral artery, simulating the ischaemic and hypoxic injury caused by the temporary clamping of large vessels in clinical surgery.

Diabetes is characterized by chronic systemic inflammation that causes an immune response and then leads to microvascular dysfunction [5]. Studies have shown that diabetes worsens neurological damage after stroke [29, 30]. Our research also confirmed that diabetes exacerbated CIRI and worsened ischaemic stroke outcomes. Neuroinflammation is worth mentioning in CIRI. The activation of danger-associated molecular patterns (DAMPs) after stroke triggers the production of various cytokines, which cause neuroinflammation and result in neuronal damage. Moreover, CIRI activates microglia, causing the removal of dead neurons and the restoration of nerve function [31]. Overactivated microglia release cytotoxins in the central nervous system, leading to neuronal death [32]. MCP-1 is a classic chemokine of microglial migration. Our data showed microglial recruitment in the ischaemic penumbra. Additionally, MCP-1 and TNF-*α* expression was significantly increased, which indicated severe inflammation in diabetic stroke.

The TLR4 signalling pathway is one of the main immune mechanisms that enhances inflammation-mediated brain injury after stroke [33]. The expression of TLR4 is increased in the immune cells of diabetic patients [34] and is probably activated by hyperglycaemia [35]. In addition, an imbalance in intestinal flora in patients with diabetes may cause high expression of TLR4 [36]. TLR4 activation is positively correlated with cerebral infarct volume, which may underlie the association of TLR4 expression with worse prognosis after stroke [37]. Our study found that diabetic ischaemic stroke increased the expression of TLR4, its adaptor MyD88 and its downstream protein phospho-NF-*κ*B, which indicated that TLR4 was activated in diabetic stroke. We further found that TLR4 was mainly expressed in microglia in the ischaemic penumbra of diabetic mice. A previous study showed that activated TLR4 can promote NLRP3 inflammasome activation, which promotes excessive inflammation [38, 39]. Correspondingly, our study showed that the NLRP3 inflammasome was also activated in the microglia of diabetic mice with CIRI.

Perioperative stroke is defined as cerebral infarction that occurs within 30 days after surgery. In surgeries, such as heart operation and carotid endarterectomy, CBF is usually temporarily blocked or reduced, resulting in iatrogenic temporary cerebral ischaemia [40]. In addition, the incidence of stroke after noncardiac surgery is as high as 2% [41]. Thus, it is very important to use appropriate anaesthetic drugs for clinical anaesthesia to prevent CIRI. Inhaled anaesthetics have been shown to be neuroprotective in ischaemic brain injury. Isoflurane, sevoflurane, and desflurane, which are often used in the clinic, have similar potential neuroprotective mechanisms, and there are no differences in their neuroprotective effects [42]. ISO is a common inhaled anaesthetic used for clinical anaesthesia that has the dual properties of neurotoxicity and neuroprotection; the effects of ISO are based on the exposure concentration and duration, as well as the brain age [43]. Low-dose and short-term exposure to ISO has shown anti-CIRI properties in vitro and in vivo [8, 44–46]. However, it is unclear whether ISO exerts neuroprotective effects in stroke complicated with diabetes. In this study, we demonstrated that ISO had neuroprotective properties. The inhibition of TLR4 may be a potential mechanism by which ISO exerts its neuroprotective effects [47–49]. We found that ISO inhibited TLR4 signalling in diabetic model mice after stroke. TAK-242 (also called CLI-095) is a selective TLR4 antagonist that blocks the TLR4 signalling pathway in mice [50]. We observed that the effect of ISO was similar to that of TAK-242. Inhibition of the NLRP3 inflammasome has been demonstrated to be a sufficient treatment for diabetic stroke [51]. Our data showed that ISO can interfere with the TLR4-NLRP3 signalling pathway in microglia in diabetic stroke. Finally, we administered the TLR4-specific agonist CRX-527 to elucidate the mechanism underlying the neuroprotective effects of ISO. We found that CRX-527 increased the cerebral infarct volume, worsened the neurobehavioural scores, and reversed the neuroprotective effect of ISO. In summary, our results indicated that the neuroprotective effects of ISO are mediated by the TLR4-NLRP3 signalling pathway.

Interestingly, ISO also inhibited autophagy in diabetic stroke. Autophagy is an important cellular process that maintains intracellular homeostasis and survival. However, stroke-induced excessive autophagy is considered harmful (10). We assessed the autophagic flux by measuring increases in LC3B, p62, and Beclin-1 expression after diabetic stroke. ISO reduced LC3B and Beclin-1 expression after stroke, indicating that the production of autophagosomes was decreased. The increase in p62 expression was inhibited by ISO, indicating that the synthesis of autophagosomes and lysosomes was increased. Additionally, this process is likely to occur in microglia. However, the changes in the autophagic flux induced by stroke remain complex, and we have not conducted in-depth research on the relationship between the neuroprotective properties of ISO and the autophagic flux, which is a limitation of this study and requires further exploration.

In summary, our research revealed important evidence of a close relationship between diabetes and stroke. We demonstrated that ISO reduced CIRI in the context of diabetes by inhibiting the TLR4-NLRP3 signalling pathway. ISO may be an important and clinically relevant drug for patients who need to undergo general anaesthesia but are prone to perioperative ischaemic stroke.

## Figures and Tables

**Table 1:** The scoring criteria.

Scores	Features
0	Normal activity without neurological impairment
1	Inability to fully extend the contralateral forelimb in the tail lifting experiment
2	Rotation to the opposite side when walking but normal posture at rest
3	Dumping to the opposite side at rest
4	No autonomic activity and disruption of consciousness

## Data Availability

The data used to support the findings of this study are available from the corresponding author upon reasonable request.

## Supplementary Materials

Supplementary Materials S1: antibody information in this experiment. Supplementary Figure S1: western blot images of TLR4, p-NF-*κ*B p65, and NLRP3 inflammasome-related proteins in non-DM and DM mice.

## References

[1] Wu S., Wu B., Liu M. (2019). Stroke in China: advances and challenges in epidemiology, prevention, and management. *Lancet Neurology*.

[2] Campbell B. C. V., Khatri P. (2020). Stroke. *Stroke. Lancet*.

[3] Gaudino M., Benesch C., Bakaeen F. (2020). Considerations for reduction of risk of perioperative stroke in adult patients undergoing cardiac and thoracic aortic operations: a scientific statement from the American Heart Association. *Circulation*.

[4] Newman J., Wilcox T., Smilowitz N., Berger J. (2018). Influence of diabetes on trends in perioperative cardiovascular events. *Diabetes Care*.

[5] van Sloten T. T., Sedaghat S., Carnethon M. R., Launer L. J., Stehouwer C. D. A. (2020). Cerebral microvascular complications of type 2 diabetes: stroke, cognitive dysfunction, and depression. *The Lancet Diabetes and Endocrinology*.

[6] Raub D., Platzbecker K., Grabitz S. D. (2021). Effects of volatile anesthetics on postoperative ischemic stroke incidence. *Journal of the American Heart Association*.

[7] Altay O., Suzuki H., Hasegawa Y., Ostrowski R. P., Tang J., Zhang J. H. (2014). Isoflurane on brain inflammation. *Neurobiology of Disease*.

[8] Zhang H., Xiong X., Liu J. (2016). Emulsified isoflurane protects against transient focal cerebral ischemia injury in rats via the PI3K/Akt signaling pathway. *Anesthesia and Analgesia*.

[9] Yan W., Chen Z., Chen J., Chen H. (2016). Isoflurane preconditioning protects rat brain from ischemia reperfusion injury via up-regulating the HIF-1alpha expression through Akt/mTOR/s6K activation. *Cellular and Molecular Biology (Noisy-le-Grand, France)*.

[10] Shi K., Tian D. C., Li Z. G., Ducruet A. F., Lawton M. T., Shi F. D. (2019). Global brain inflammation in stroke. *Lancet Neurology*.

[11] Hyakkoku K., Hamanaka J., Tsuruma K. (2010). Toll-like receptor 4 (TLR4), but not TLR3 or TLR9, knock-out mice have neuroprotective effects against focal cerebral ischemia. *Neuroscience*.

[12] Lin H. B., Lin Y. H., Zhang J. Y. (2021). NLRP3 Inflammasome: a potential target in isoflurane pretreatment alleviates stroke-induced retinal injury in diabetes. *Frontiers in Cellular Neuroscience*.

[13] Lu J., Wang X., Feng Z., Chen Y., Wen D., Liu Z. (2021). The protective effect of isoflurane pretreatment on liver IRI by suppressing noncanonical pyroptosis of liver macrophages. *International Immunopharmacology*.

[14] Sun M., Deng B., Zhao X. (2015). Isoflurane preconditioning provides neuroprotection against stroke by regulating the expression of the TLR4 signalling pathway to alleviate microglial activation. *Scientific Reports*.

[15] Li C., Che L. H., Ji T. F., Shi L., Yu J. L. (2017). Effects of the TLR4 signaling pathway on apoptosis of neuronal cells in diabetes mellitus complicated with cerebral infarction in a rat model. *Scientific Reports*.

[16] Zhou R., Yazdi A. S., Menu P., Tschopp J. (2011). A role for mitochondria in NLRP3 inflammasome activation. *Nature*.

[17] Hong P., Gu R. N., Li F. X. (2019). NLRP3 inflammasome as a potential treatment in ischemic stroke concomitant with diabetes. *Journal of Neuroinflammation*.

[18] Hong P., Li F. X., Gu R. N. (2018). Inhibition of NLRP3 inflammasome ameliorates cerebral ischemia-reperfusion injury in diabetic mice. *Neural Plasticity*.

[19] Ajoolabady A., Wang S., Kroemer G. (2021). Targeting autophagy in ischemic stroke: from molecular mechanisms to clinical therapeutics. *Pharmacology & Therapeutics*.

[20] Seay M. D., Dinesh-Kumar S. P. (2007). Autophagy takes its TOLL on innate immunity. *Cell Host & Microbe*.

[21] Lin H. B., Wei G. S., Li F. X. (2020). Macrophage-NLRP3 inflammasome activation exacerbates cardiac dysfunction after ischemic stroke in a mouse model of diabetes. *Neuroscience Bulletin*.

[22] Yuan L., Li Y., Li H., Lu H., Tong S. (2015). Intraoperative laser speckle contrast imaging improves the stability of rodent middle cerebral artery occlusion model. *Journal of Biomedical Optics*.

[23] Yang J., Liu H., Han S. (2020). Melatonin pretreatment alleviates renal ischemia-reperfusion injury by promoting autophagic flux via TLR4/MyD88/MEK/ERK/mTORC1 signaling. *The FASEB Journal*.

[24] Hua F., Tang H., Wang J. (2015). TAK-242, an antagonist for Toll-like receptor 4, protects against acute cerebral ischemia/reperfusion injury in mice. *Journal of Cerebral Blood Flow and Metabolism*.

[25] Kapadia R., Tureyen K., Bowen K. K., Kalluri H., Johnson P. F., Vemuganti R. (2006). Decreased brain damage and curtailed inflammation in transcription factor CCAAT/enhancer binding protein *β* knockout mice following transient focal cerebral ischemia. *Journal of Neurochemistry*.

[26] Xu Y., Jagannath C., Liu X. D., Sharafkhaneh A., Kolodziejska K. E., Eissa N. T. (2007). Toll-like receptor 4 is a sensor for autophagy associated with innate immunity. *Immunity*.

[27] Wu A. G., Zhou X. G., Qiao G. (2021). Targeting microglial autophagic degradation in NLRP3 inflammasome-mediated neurodegenerative diseases. *Ageing Research Reviews*.

[28] Ma R., Xie Q., Li Y. (2020). Animal models of cerebral ischemia: a review. *Biomedicine & Pharmacotherapy*.

[29] Grisotto C., Taïlé J., Planesse C. (2021). High-fat diet aggravates cerebral infarct, hemorrhagic transformation and neuroinflammation in a mouse stroke model. *International Journal of Molecular Sciences*.

[30] MacDougall N. J., Muir K. W. (2011). Hyperglycaemia and infarct size in animal models of middle cerebral artery occlusion: systematic review and meta-analysis. *Journal of Cerebral Blood Flow and Metabolism*.

[31] Xiong X. Y., Liu L., Yang Q. W. (2016). Functions and mechanisms of microglia/macrophages in neuroinflammation and neurogenesis after stroke. *Progress in Neurobiology*.

[32] Leitner G. R., Wenzel T. J., Marshall N., Gates E. J., Klegeris A. (2019). Targeting toll-like receptor 4 to modulate neuroinflammation in central nervous system disorders. *Expert Opinion on Therapeutic Targets*.

[33] Famakin B. M., Vemuganti R. (2020). Toll-like receptor 4 signaling in focal cerebral ischemia: a focus on the neurovascular unit. *Molecular Neurobiology*.

[34] Alibashe-Ahmed M., Brioudes E., Reith W., Bosco D., Berney T. (2019). Toll-like receptor 4 inhibition prevents autoimmune diabetes in NOD mice. *Scientific Reports*.

[35] Singh A., Boden G., Rao A. K. (2015). Tissue factor and Toll-like receptor (TLR)4 in hyperglycaemia-hyperinsulinaemia. Effects in healthy subjects, and type 1 and type 2 diabetes mellitus. *Thrombosis and Haemostasis*.

[36] Singer-Englar T., Barlow G., Mathur R. (2019). Obesity, diabetes, and the gut microbiome: an updated review. *Expert Review of Gastroenterology & Hepatology*.

[37] Parada E., Casas A., Palomino-Antolin A. (2019). Early toll-like receptor 4 blockade reduces ROS and inflammation triggered by microglial pro-inflammatory phenotype in rodent and human brain ischaemia models. *British Journal of Pharmacology*.

[38] Gaidt M. M., Ebert T. S., Chauhan D. (2016). Human monocytes engage an alternative inflammasome pathway. *Immunity*.

[39] Alishahi M., Farzaneh M., Ghaedrahmati F., Nejabatdoust A., Sarkaki A., Khoshnam S. E. (2019). NLRP3 inflammasome in ischemic stroke: as possible therapeutic target. *International Journal of Stroke*.

[40] Apostolakis E., Akinosoglou K. (2008). The methodologies of hypothermic circulatory arrest and of antegrade and retrograde cerebral perfusion for aortic arch surgery. *Annals of Thoracic and Cardiovascular Surgery*.

[41] Vlisides P. E., Moore L. E. (2021). Stroke in surgical patients. *Anesthesiology*.

[42] Matchett G. A., Allard M. W., Martin R. D., Zhang J. H. (2009). Neuroprotective effect of volatile anesthetic agents: molecular mechanisms. *Neurological Research*.

[43] Neag M., Mitre A., Catinean A., Mitre C. (2020). An overview on the mechanisms of neuroprotection and neurotoxicity of isoflurane and sevoflurane in experimental studies. *Brain Research Bulletin*.

[44] Sakai H., Sheng H., Yates R. B., Ishida K., Pearlstein R. D., Warner D. S. (2007). Isoflurane provides long-term protection against focal cerebral ischemia in the rat. *Anesthesiology*.

[45] Mutoh T., Mutoh T., Sasaki K. (2016). Isoflurane postconditioning with cardiac support promotes recovery from early brain injury in mice after severe subarachnoid hemorrhage. *Life Sciences*.

[46] Xu X., Kim J. A., Zuo Z. (2008). Isoflurane preconditioning reduces mouse microglial activation and injury induced by lipopolysaccharide and interferon-*γ*. *Neuroscience*.

[47] Xiang H. F., Cao D. H., Yang Y. Q. (2014). Isoflurane protects against injury caused by deprivation of oxygen and glucose in microglia through regulation of the Toll-like receptor 4 pathway. *Journal of Molecular Neuroscience*.

[48] Okuno T., Koutsogiannaki S., Hou L. (2019). Volatile anesthetics isoflurane and sevoflurane directly target and attenuate Toll-like receptor 4 system. *The FASEB Journal*.

[49] Abdul Y., Abdelsaid M., Li W. (2019). Inhibition of Toll-like receptor-4 (TLR-4) improves neurobehavioral outcomes after acute ischemic stroke in diabetic rats: possible role of vascular endothelial TLR-4. *Molecular Neurobiology*.

[50] Fellner A., Barhum Y., Angel A. (2017). Toll-like Receptor-4 inhibitor TAK-242 attenuates motor dysfunction and spinal cord pathology in an amyotrophic lateral sclerosis mouse model. *International Journal of Molecular Sciences*.

[51] Ward R., Li W., Abdul Y. (2019). NLRP3 inflammasome inhibition with MCC950 improves diabetes-mediated cognitive impairment and vasoneuronal remodeling after ischemia. *Pharmacological Research*.

