# Supplementary material for: Isoflurane Attenuates Cerebral Ischaemia–Reperfusion Injury via the TLR4-NLRP3 Signalling Pathway in Diabetic Mice
Source: Oxid Med Cell Longev. 2022 Apr 4;2022:2650693. doi: 10.1155/2022/2650693 (PMC9001073; doi:10.1155/2022/2650693)
Supplement: Supplementary Materials — S1: antibody information in this experiment. Supplementary Figure S1: western blot images of TLR4, p-NF-κB p65, and NLRP3 inflammasome-related proteins in non-DM and DM mice. [file 2650693.f1.docx]

**Supplementary information**

**Table S1. Antibody information**

| Antibody | Catalogue number | Producer | WB | IHC | IF |
| --- | --- | --- | --- | --- | --- |
| TLR4 | MA5-16216 | Invitrogen, USA | 1:1000 | 1:200 | 1:200 |
| MyD88 | AF5195 | Affinity, USA | 1:1000 |  |  |
| p-P65 | ab183559 | Abcam, USA | 1:1000 |  |  |
| NLRP3 | PA579740 | Invitrogen, USA | 1:1000 |  | 1:400 |
| IL-1β | 12703 | CST, USA | 1:1000 |  |  |
| MCP-1 | PA534505 | Invitrogen, USA | 1:1000 |  |  |
| TNF-α | st-52746 | SANATA CRUZ, USA | 1:1000 |  |  |
| LC3B | PA1-46286 | Invitrogen, USA | 1:1000 |  | 1:400 |
| p62 | 5114 | CST, USA | 1:1000 |  |  |
| Beclin-1 | 3738 | CST, USA | 1:1000 |  |  |
| α-Tubulin | RM2007 | Ray Antibody, China | 1:1000 |  |  |
| β-actin | RM2001 | Ray Antibody, China | 1:1000 |  |  |
| IBA-1 | ab178846 | Abcam, USA | 1:2000 | 1:2000 | 1:2000 |
| Goat anti rabbit | a-11034 | Seymour Fisher, USA |  |  | 1:1000 |
| Goat anti mouse | a-11032 | Seymour Fisher, USA |  |  | 1:1000 |

**Supplementary figure S1**


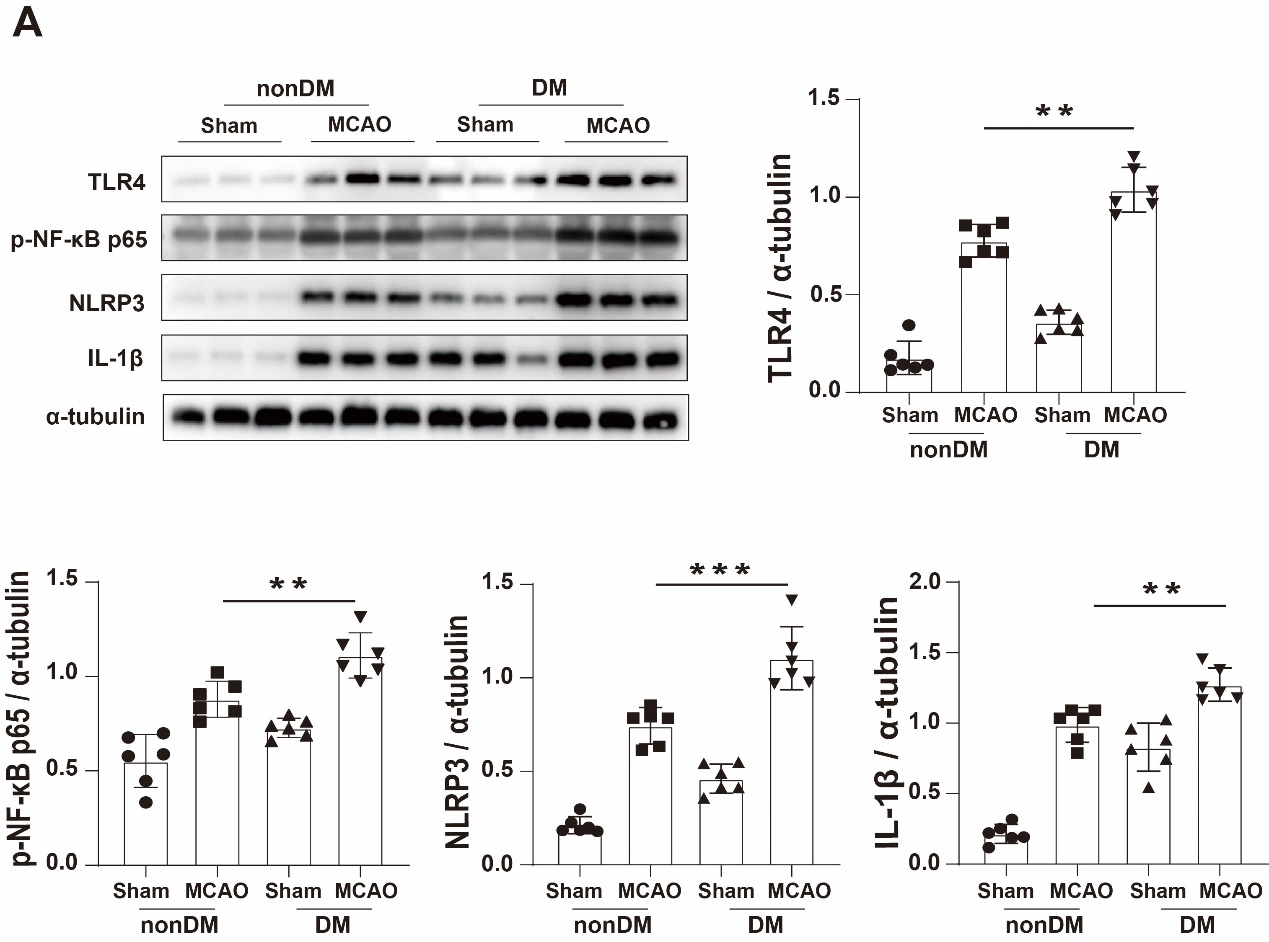


**Supplementary Fig. S1** **Cerebral ischaemia-reperfusion induced more inflammatory under diabetes than non-diabetes.**

(A) Top left, western blot images of TLR4, p-NF-κB p65 and NLRP3 inflammasome-related proteins (n=3 in each group). Top right, the expression of TLR4. Bottom, the expression of p-NF-κB p65 (left) and NLRP3 (middle) and IL-1β (right, n=6 in each group). ***P* < 0.01; ****P* < 0.001.
